# IFITM Genes, Variants, and Their Roles in the Control and Pathogenesis of Viral Infections

**DOI:** 10.3389/fmicb.2018.03228

**Published:** 2019-01-08

**Authors:** Xuesen Zhao, Jiarui Li, Cheryl A. Winkler, Ping An, Ju-Tao Guo

**Affiliations:** ^1^Institute of Infectious Disease, Beijing Ditan Hospital, Capital Medical University, Beijing, China; ^2^Beijing Key Laboratory of Emerging Infectious Disease, Beijing, China; ^3^Basic Research Laboratory, Frederick National Laboratory for Cancer Research, National Cancer Institute, Leidos Biomedical Research, Inc., Frederick, MD, United States; ^4^Baruch S. Blumberg Institute, Hepatitis B Foundation, Doylestown, PA, United States

**Keywords:** host susceptibility, interferon-induced transmembrane proteins, IFITM, single nucleotide polymorphisms, viral infection

## Abstract

Interferon-induced transmembrane proteins (IFITMs) are a family of small proteins that localize in the plasma and endolysosomal membranes. IFITMs not only inhibit viral entry into host cells by interrupting the membrane fusion between viral envelope and cellular membranes, but also reduce the production of infectious virions or infectivity of progeny virions. Not surprisingly, some viruses can evade the restriction of IFITMs and even hijack the antiviral proteins to facilitate their infectious entry into host cells or promote the assembly of virions, presumably by modulating membrane fusion. Similar to many other host defense genes that evolve under the selective pressure of microorganism infection, IFITM genes evolved in an accelerated speed in vertebrates and many single-nucleotide polymorphisms (SNPs) have been identified in the human population, some of which have been associated with severity and prognosis of viral infection (e.g., influenza A virus). Here, we review the function and potential impact of genetic variation for IFITM restriction of viral infections. Continuing research efforts are required to decipher the molecular mechanism underlying the complicated interaction among IFITMs and viruses in an effort to determine their pathobiological roles in the context of viral infections *in vivo*.

Interferon-induced transmembrane proteins (IFITMs) are a family of small proteins that can be found in single cell organisms and are evolutionally conserved across vertebrates (Siegrist et al., 2011; Zhang et al., 2012). The human IFITM family comprises five members, including immune-related IFITM1, IFITM2, and IFITM3, as well as IFITM5 and IFITM10 with no known role in immunity. As key host defense genes, IFITMs evolved under the selective pressure of microorganism infection (Compton et al., 2016). IFITM proteins are involved in many aspects of virus–host interaction and play important roles in viral pathogenesis. Among the number of single-nucleotide polymorphisms (SNPs) in *IFITM3* gene that have been identified in human populations, several are associated with disease severity and prognosis of influenza A virus (IAV) and other viral infections (Everitt et al., 2012; Zhang et al., 2015; Xu-Yang et al., 2016; Allen et al., 2017). Mechanistically, these SNPs either alter the expression of IFITM3 or result in expression of N-terminally truncated IFITM3 isoform, Δ21-IFITM3, with reduced antiviral activity against different viruses (Everitt et al., 2012; Allen et al., 2017). In this review, we will summarize the findings on human IFITM structural features related with antiviral activity, and impact of genetic variation on IFITM antiviral function in the control and pathogenesis of viral infections in humans.

## IFITMs Restrict a Broad Spectrum of Viruses in Cultured Cells and *In Vivo*

To date, IFITMs have been shown to inhibit the infection of enveloped RNA viruses from 9 viral families (Perreira et al., 2013), non-enveloped RNA viruses, e.g., reovirus (Anafu et al., 2013) and foot-and-mouth disease virus (Xu et al., 2014), and several DNA viruses (Li et al., 2018). IFITMs efficiently inhibit a number of medically important human pathogenic viruses, including IAV (Brass et al., 2009; Bailey et al., 2012), dengue virus (DENV) (Brass et al., 2009; Jiang et al., 2010), West Nile virus (WNV) (Brass et al., 2009; Jiang et al., 2010), Zika Virus (ZIKV) (Savidis et al., 2016), Ebola virus (EBOV) (Huang et al., 2011; Wrensch et al., 2015), Marburg virus (MARV) (Huang et al., 2011), severe acute respiratory syndrome coronavirus (SARS-CoV) (Huang et al., 2011), Rift Valley fever virus (RVFV) (Mudhasani et al., 2013), Hantaan virus (HTNV) (Mudhasani et al., 2013; Xu-Yang et al., 2016), hepatitis C virus (HCV) (Wilkins et al., 2013), and human immunodeficiency virus (HIV) (Lu et al., 2011; Compton et al., 2014; Yu et al., 2015; Compton et al., 2016; Foster et al., 2016; Chesarino et al., 2017; Tartour et al., 2017; Wang et al., 2017). *In vivo* studies in *IFITM3* knockout mice demonstrate the critical role of IFITM3 in restricting infection and reducing disease severity of infection by IAV (Bailey et al., 2012; Everitt et al., 2012), WNV (Gorman et al., 2016), Chikungunya virus and Venezuelan equine encephalitis virus (Poddar et al., 2016), and respiratory syncytial virus (Everitt et al., 2013). IFITM3 in mice not only protects lung epithelia cells from IAV infection, but it was also shown to restricts IAV infection of lung dendritic cells, which traffic to lymph nodes to prime CD8^+^ T cell anti-viral response (Infusini et al., 2015). Moreover, lung resident memory CD8^+^ T cells in mice were programmed to retain IFITM3 expression, facilitating their survival and protection from viral infection during subsequent exposures (Wakim et al., 2013).

## IFITMs Mainly Restrict Virus Infection at Cell Entry

IFITM proteins localize at the plasma membrane as well as the membranes of endocytic vesicles and lysosomes (Bailey et al., 2014). IFITMs can also be incorporated into envelope membranes of many viruses (Yu et al., 2015; Tartour et al., 2017). Emerging evidence suggests that IFITM proteins on both viral and cellular membranes can restrict the infectious entry of diverse envelope viruses by inhibiting viral fusion at cell plasma or endolysosomal membranes, but IFITM does not impede endocytosis of virions into cells (Weidner et al., 2010; Li et al., 2013; Perreira et al., 2013; Compton et al., 2014; Desai et al., 2014). As extensively discussed in a previous review (Perreira et al., 2013), the potency of IFITM restriction of viral cell entry is generally correlated to the co-localization of IFITM proteins at the sites of viral fusion. For instance, IFITM1 more efficiently restricts the viruses that enter the cytoplasm *via* direct fusion with plasma membrane *or via Rab-5 positive early endosomes*, whereas IFITM3 more efficiently inhibits viruses that enter *via* Rab7-positive late endosomes or lysosomes. This rule is highlighted by the finding that mutation of IFITM3 endocytic signal results in its cell surface accumulation and gains a function to restrict the infection of human parainfluenza virus 3 (HPIV-3), which enters cells *via* direct fusion with the plasma membrane (Rabbani et al., 2016; Zhao et al., 2018). Another example is that the sensitivity of IAVs to IFITM3 appears to depend on the pH value at which the IAV hemagglutinin triggers membrane fusion and thus the endocytic compartments where the membrane fusion take place (Gerlach et al., 2017). However, exceptions of this rule do exist. For instance, it remains to know how Moloney leukemia virus (MLV) and Sendai virus that fuse at cell plasma membrane (Brass et al., 2009; Hach et al., 2013) as well as Lassa fever virus (LASV) and lymphocytic choriomeningitis virus (LCMV) that fuse at Rab7-positive late endosomes (Brass et al., 2009; Mudhasani et al., 2013) to escape IFITM1 and IFITM3 restriction, respectively.

The mechanism of IFITM inhibition of viral fusion and cell entry is not yet resolved. One study reported that IFITM inhibited Jaagsiekte sheep retrovirus envelope and IAV hemagglutinin fusion of viral envelope to cellular membranes prior to coalescence of lipid-bilayers, a process known as hemifusion (Li et al., 2013). However, another study using a direct virus-cell fusion assay in viable cells to investigate IAV entry found that overexpression of IFITM3 protein in late endosomes did not alter lipid mixing, but rather inhibited the release of viral contents into the cytoplasm, suggesting that IFITM3 inhibits the transition from hemifusion to full fusion of the respective lipid membranes (Desai et al., 2014). Others studies suggested that IFITM multimerization decreases membrane flexibility by altering membrane curvature, with the consequence of interruption of the virus-cell membrane fusion (John et al., 2013; Lin et al., 2013). Another group reported that IFITM expression increased cholesterol content in the endosome or lysosome *via* an interaction with vesicle-associated membrane protein-associated protein A (VAPA), which abrogated endolysosomal fusion with the viral envelop (Amini-Bavil-Olyaee et al., 2013). However, this later observation was not confirmed by other studies (Desai et al., 2014; Wrensch et al., 2014). In addition, IFITMs were reported to affect the trafficking of vacuolar ATPase (v-ATPase), implying that IFITMs may indirectly restrict viral entry by modulating endosomal acidity (Wee et al., 2012). It is also well documented that the antiviral potency of IFITM proteins varies among different cell types (Huang et al., 2011; Zhao et al., 2018), suggesting that IFITMs work together with other cellular proteins to modulate viral fusion. In support of this notion, zinc metallopeptidase STE24 (ZMPSTE24), a transmembrane metalloprotease localized in the inner nuclear membrane and cytoplasmic organelles, had been identified as a downstream partner of IFITM3 to restrict the entry of enveloped RNA and DNA viruses (Fu et al., 2017).

In addition to the protective function of IFITM proteins to reduce viral infection of host cells, IFITM proteins, particularly IFITM2 and 3, can lead to the production of virions that package IFITMs and display reduced entry into target cells (Compton et al., 2014; Tartour et al., 2014, 2017). One study found that IFITM proteins interact with HIV envelope protein in viral producer cells to disrupt envelope protein processing and virion incorporation, which impairs virion infectivity (Yu et al., 2015); however, another found that IFITM3 expression in producing cells did not affect the amount of envelope protein incorporation into progeny HIV-1 virions (Appourchaux et al., 2018). Both studies reported that the level of IFITM protein incorporation into progeny virions does not correlate with the extent of infectivity reduction.

Moreover, recent studies have also shown that IFITMs can modulate viral infection and pathogenesis *via* mechanisms that are not directly related to the restriction of virus entry. For example, given the resistance of human papillomaviruses (HPV) to IFITMs, HPV infection of keratinocytes inhibits the expression of IFITM1 and RIPK3 to escape from IFN-γ and TNF-α-mediated antiproliferation and necroptosis, which is essential for establishing a persistent infection (Ma et al., 2016). In addition, it was demonstrated in IFITM3 knockout mice that IFITM3 limited murine CMV (MCMV) pathogenesis without directly preventing virus replication (Stacey et al., 2017). Instead, IFITM3 contributed to the antiviral cellular immunity by abrogating inflammatory cytokine-driven lymphopenia including apoptosis-independent NK cell death and T cells depletion (Stacey et al., 2017).

## Viral Counteraction for Evading IFITMs

Not surprisingly, in the arms race between pathogen and host, many viruses have evolved strategies to evade the antiviral function of host restriction proteins. For instance, transmitted founder HIV-1 strains establishing *de novo* infection are generally capable of evading IFITM restriction (Foster et al., 2016; Wang et al., 2017). Mutations allow the virus to escape adaptive immune responses, and/or switches in the HIV-1 co-receptor tropism from CCR5 to CXCR4 increases viral sensitivity to inhibition by IFITM2 and IFITM3 in endosomal compartments (Foster et al., 2016). Moreover, IAV facilitates its infection by activating p53 or degrading eukaryotic translation initiation factor 4B (eIF4B) to inhibit the expression of IFITM proteins (Wang S. et al., 2014; Wang et al., 2018). In contrast, human coronavirus OC43 (HCoV-OC43) and human cytomegalovirus (HCMV) hijack IFITM3 to promote its infectious entry and progeny virions assembly, respectively (Zhao et al., 2014; Xie et al., 2015).

## Structure and Polymorphism of IFITM Gene Locus

**FIGURE 1:**
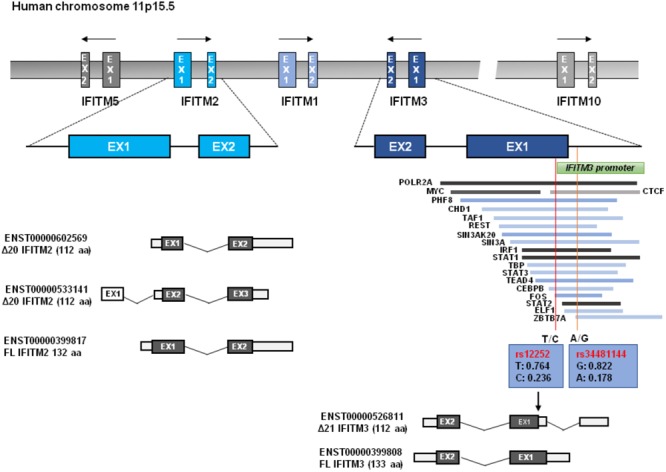
*IFITM* gene structure. Human *IFITM* genes located in chromosome 11p15.5 are indicated, and the exons of immunity-related *IFITM* genes (*IFITM1, IFITM2*, and *IFITM3*) are indicated in blue color. The putative transcripts of *IFITM2* and *IFITM3* are also shown below. The transcription factor binding regions derived from UCSC Genome Browser are shown, which is verified by Chip-seq from the 161 proteins tested through ENCODE; GRCh37/hg19. Two red vertical lines across transcription factor binding sites represent the Influenza associated SNPs of rs12252 and rs34481144 with allele frequencies, respectively. N-terminally truncated IFITM3 isoform (Δ21 IFITM3) that is presumably generated by rs12252 is indicated.

The human *IFITM* locus, approximately 18kb long, is located on chromosome 11 and comprises five genes: *IFITM1, IFITM2, IFITM3, IFITM5*, and *IFITM10* (Figure 1). As an IFN-stimulated gene (ISG), *IFITM1, IFITM2, IFITM3* genes each has an interferon stimulated response element (ISRE) in its promoter region besides the additional gamma-activated sequence (GAS) in the promoter region of *IFITM1* gene (Siegrist et al., 2011). However, the protein expression of IFITM5 and IFITM10 are not induced by IFNs (Zhang et al., 2012). Though IFITM1-3 proteins are ubiquitously expressed in human tissues in the absence of IFN induction, they can be robustly up-regulated by all three types of IFNs (Zhao et al., 2014). The *IFITM3* promoter has binding sites for dozens of transcription factors, including POLR2A, MYC, ELF1, PHF8, CHD1, TAF1, REST, SIN3AK20, SIN3A, IRF1, STAT1, TBP, STAT3, STAT2, ZBTB7A, and CTCF, some of which may affect IFITM3 expression (Allen et al., 2017).

As illustrated in Figure 1, all *IFITM* genes contain two coding exons interspersed by one intron. Both *IFITM2* and *IFITM3* were predicated to encode a wild typed full-length form and a truncated isoform with 20/21 amino acid residues deletion from N-terminus. Most vertebrate animals have two or more IFITM genes. In many primate species, gene duplication, and divergence of *IFITM3* has been identified (Zhang et al., 2012; Compton et al., 2016). *IFITM* locus in many modern primate species contains multiple copies of *IFITM3*-like genes due to gene duplication (Compton et al., 2016). For instance, Marmoset, Macaque, and Africa Green Monkey (AGM) each has five, six and eight copies of *IFITM3 genes*, respectively (Compton et al., 2016). Comparative genomics studies indicate that *IFITM3* is the most ancient member in the *IFITM* family and *IFITM2* emerges as a rather recent genetic event in human, chimpanzee, and gorilla (Compton et al., 2016). The multiplicity and diversity of *IFITM2/3* indicates that there is a positive selection in *IFITM* evolution, which is consistent with their role in restricting pathogen invasion (Compton et al., 2016).

## Structural Function Relationship of IFITM Proteins

**FIGURE 2:**
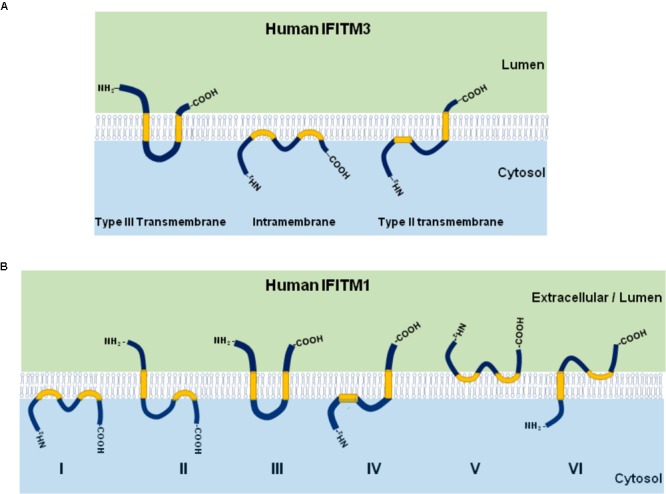
Illustration of IFITM protein membrane topology. The three possible membrane topologies of IFITM3 **(A)** and six possible membrane topological forms of IFITM1 **(B)** are presented. The two transmembrane (or intramembrane) regions were highlighted in yellow.

IFITM proteins have several topologies that may affect their function. As shown in Figure 2A, although IFITM3 may adopt three different membrane topologies (Bailey et al., 2014), it exists predominantly as a type II transmembrane protein with the N-terminus in cytosol and the short C-terminus exposed to cellular exterior or in the lumen of endolysosome (Bailey et al., 2013). Although IFITM1 was also reported to predominately adopt a type II transmembrane topology, other membrane topologies may exist (Figure 2B; Li et al., 2015).

**FIGURE 3:**
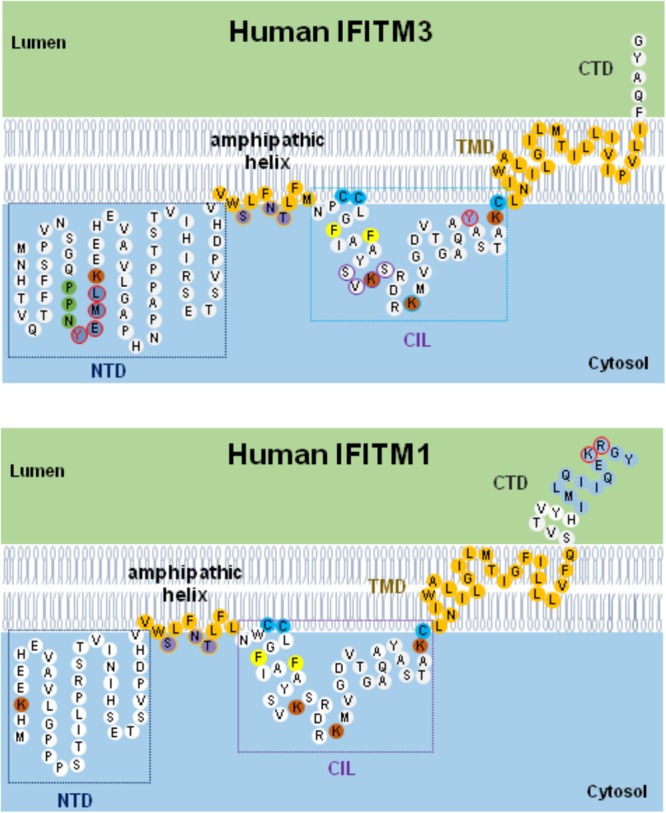
Schematic diagram of IFITM1/3 topology and structural determinants essential for their modulation of viral entry. Five structural domains, including the N-terminal domain (NTD), amphipathic helix domain (AHD), intracellular loop (CIL), transmembrane domain (TMD), and C-terminal domain (CTD), are illustrated. Two membrane-associated domains (AHD and TMD) are embodied in light orange. In IFITM3, three hydrophilic residues (S61, N64, and T65) in amphipathic helix are labeled in violet. In NTD domain, ^20^YEML^23^ motif required for IFITM3 endocytosis are depicted in blue with red circle and ^17^PPNY^20^ motif recruiting NEDD4 E3 ligase are shown in green. In CIL domain, ^81^SVKS^84^ motif required for IFITM3 to inhibit IAV, DENV, and LLOV infection is indicated with purple circle. The residues with post-translational modification, including phosphorylation (Y20 and Y99 labeled with red circle), palmitoylation (C71, C72, and C105 marked with light blue), and ubiquitination (highlighted in orange), are indicated. In addition, two residues (F75 and F78) essential for oligomerization are marked with yellow. In IFITM1, C-terminal 12-aa residues critical for modulating human coronaviruses entry is indicated in blue, and ^122^KR^123^ dibasic motif constituted as IFITM1 sorting signal is depicted with red circle.

As depicted in Figure 3, IFITMs consist of intramembrane (IMD) and transmembrane (TMD) domains separated by an intracellular loop (CIL) and variable N and C terminal domains (NTD and CTD), respectively (Bailey et al., 2013, 2014; Chesarino et al., 2017). While the NTD and CTD are highly variable in length and sequence among IFITM orthologs and paralogs, the canonical CD225 domain spanning IMD to CIL domains is evolutionally more conserved (Bailey et al., 2014). Critical structure motifs and amino acid residues undergoing post-translational modifications required for IFITM oligomerization as well as their biological and antiviral functions are discussed below.

### NTD of IFITM2 and IFITM3

Compared to IFITM1, IFITM2 and IFITM3 have N-terminal 20-aa or 21-aa extension which was previously regarded to be absent in rs12252-C encoding IFITM3 isoform. Although deletion of this N-terminal 21-aa region significantly impaired its ability to inhibit the infection by IAV, VSV, and DENV, the deletion apparently did not affect its ability to enhance HCoV-OC43pp infection (Weidner et al., 2010; John et al., 2013). Interestingly, Δ21-IFITM3 enhanced inhibition of HIV-1 fusion (Compton et al., 2016). Moreover, Δ20-IFITM2, a N-terminal 20-aa truncated isoform of IFITM2, which is derived from an alternatively initiated RNA transcript (Figure 1), demonstrated a more potent suppression on HIV-1 infection than that by full-length wild-type IFITM2 (Wu et al., 2017). In fact, a recent study argues that the Δ20-IFITM2, rather than the full-length IFITM2 and IFITM3, is the effective restriction factor of HIV-1 with CXCR4-tropism (Wu et al., 2017).

The ^20^YXXΦ^23^ motif of IFITM3 is an endocytic signal essential for endocytosis and localization of IFITM3 to endocytic vesicles and lysosomes (Jia et al., 2014). Artificial mutations of ^20^YEML^23^ motif (Y20D, Y20A, or L23A) result in an accumulation of IFITM3 in the plasma membrane and reduced inhibition of viruses that enter the cytoplasm at endocytic vesicles/lysosomes, such as IAV, human coronaviruses NL63 and -229E (Chesarino et al., 2014a; Jia et al., 2014; Williams et al., 2014; Zhao et al., 2018), but enhanced inhibition of viruses that directly enter cells at the plasma membranes, such as HPIV-3 and HIV-1 (Jia et al., 2012; Compton et al., 2016; Rabbani et al., 2016). Intriguingly, replacement of IFITM3 tyrosine (Y) 20 with either alanine (A) or aspartic acid (D) to mimic unphosphorylated or phosphorylated IFITM3 converted the antiviral protein to enhance the entry of SARS-CoV and MERS-CoV (Zhao et al., 2018). These studies suggest that the NTD, particularly ^20^YXXΦ^23^ motif, plays important roles in IFITM2/3 subcellular localization and antiviral activity. IFITM3 are phosphorylated by the protein-tyrosine kinase Fyn on tyrosine 20 (Y20), which results in its plasma membrane accumulation and decreased antiviral activity against influenza viruses (Jia et al., 2012, 2014; Chesarino et al., 2014a). This result is consistent with the mutagenesis studies of ^20^YXXΦ^23^ motif, where Y20 is part of an endocytosis signal that can be blocked by phosphorylation (Chesarino et al., 2014a). Additionally, phosphorylation of IFITM3 by Fyn and mutagenesis of Y20 also lead to decreased IFITM3 ubiquitination, suggesting modification of Y20 as an important mechanism to control IFITM3 trafficking and degradation (Chesarino et al., 2014a).

### CTD of IFITM1

Compared to IFITM2 and IFITM3, human IFITM1 has a relatively long C-terminal region of 18 amino acid residues. The ^122^KRXX^125^ motif serves as a sorting signal for IFITM1. Substitution of two basic residues ^122^KR^123^ with alanine (KR/AA) in IFITM1 reduced its distribution in LAMP1-positive lysosomes but enriched its localization in CD63-positive multivesicular bodies (Li et al., 2015). The KR/AA mutant IFITM1 executed increased activity to inhibit the infection by Jaagsiekte sheep retrovirus (JSRV) and 10A1 amphotropic murine leukemia virus (MLV) (Li et al., 2015). Interestingly, although SARS-CoV and HCoV-NL63 share the ACE2 receptor for infection of host cells, deletion of the C-terminal 3, 6, or 9 amino acids did not apparently affect the activity of IFITM1 to inhibit SARS-CoV entry but enhanced the activity of IFITM1 to inhibit the entry of HCoV-NL63 (Zhao et al., 2018). However, further deletion of the C-terminal 12, 15, or 18 amino acids significantly attenuated or even abolished the ability of IFITM1 to inhibit the entry of SARS-CoV, but did not apparently affect the entry of NL63 (Zhao et al., 2018). More strikingly, deletion of C-terminal 12-aa or 18-aa converted IFITM1 to a potent enhancer of MERS-CoV and HCoV-OC43 entry (Zhao et al., 2014, 2018). A recent mutagenesis study indicated that amino acid residues 127–132 in the CTD domain of IFITM3 differentially modulates its activities in target cell protection and negative imprinting of progeny HIV-1 infectivity (Appourchaux et al., 2018). These studies clearly indicate that the CTD of IFITMs contains multiple structure motifs that regulate the subcellular localization and antiviral functions.

### CD225 Domain

The canonical CD225 domain comprises IMD to CIL domains and is evolutionally conserved (Bailey et al., 2014). IMD domain (residues 58–80) is a hydrophobic region and possesses an amphipathic alpha helix spanning residues 59–68 (Chesarino et al., 2017). It was demonstrated recently that either deletion of this alpha helices or mutations altering amphipathicity largely impaired or even abolished IFITM antiviral activity, suggesting a critical role in IFITM antiviral function, presumably through affecting membrane physical properties (Chesarino et al., 2017). In addition, the ^81^SVKS^84^ motif residing in CIL domain is essential for IFITM3 to inhibit IAV and DENV infection, but replacement of this motif with four residues of alanine promoted cellular entry driven by Lloviu virus (LLOV) glycoprotein (Wrensch et al., 2015).

### Palmitoylation of IFITM

IFITM proteins can be palmytoylated at three cystines in CD225 domain by multiple zinc finger DHHC domain-containing palmitoyltransferases (ZDHHCs) (McMichael et al., 2017). While Cys105 localizes close to the amphipathic alpha helix in the IMD domain, Cys71 and Cys72 are adjacent to the TMD domain. Mutagenesis studies indicated that substitution of those three cysteine residues with alanine alters the IFITM3 distribution from punctate clusters in the cellular membrane to a more diffused pattern and the resulting palmitoylation-deficient mutant showed impaired or even abolished antiviral activity (Yount et al., 2010). Interestingly, when other lipid modification sites, such as myristoylation and prenylation, were introduced in IFITM NTD or CTD domains, the antiviral function of palmitoylation-deficient IFITM3 mutant could be restored. These findings imply that anchoring IFITM protein to membranes, but not structure alteration by S-palmitoylation, is important for IFITM restriction of virus entry (Yount et al., 2012; Chesarino et al., 2014b).

### Ubiquitination of IFITM

Human IFITM proteins possess four conserved lysine residues (Figure 2) that can be ubiquitinated by E3 ubiquitin ligase such as NEDD4 (Yount et al., 2012; Chesarino et al., 2015). Previous studies demonstrated that each lysine residue can be modified with mono- and poly-ubiquitination through Lys-48 and Lys-63 linkages (Yount et al., 2012). By replacing all four residues of lysine with alanine, the mutant IFITM3 demonstrated endolysosomal distribution and execute hyperactivity to restrict IAV infection than wild type (Yount et al., 2012). However, our studies demonstrated that ubi-deficient IFITM3 lost antiviral activity against all the viruses tested, including IAV (Zhao et al., 2014, 2018). The discrepancy of those studies is not clear. Importantly, ubiquitination and endocytosis of IFITM3 is required for mTOR inhibitor-induced degradation of IFITM3 (Shi et al., 2018). In addition, IFITM3 K88 can be monomethylated by lysine methyltransferase SET7 and demethylated by histone demethylase LSD1 (Shan et al., 2013, 2017). While vesicular stomatitis virus (VSV) and IAV infection increased IFITM3-K88me1 levels by promoting the interaction between IFITM3 and SET7 and disassociation from LSD1 to attenuate IFITM3 antiviral activity, IFN-α reduced IFITM3-K88me1 levels and increased its antiviral activity (Shan et al., 2013, 2017).

### Oligomerization of IFITMs

IFITM proteins function as homo- or hetero-oligomers. Two phenylalanine residues (F75 and F78) are essential for IFITM homo- and hetero-oligomerization (John et al., 2013). IFITM3 bearing F75A and F78A mutations (IFITM3/2FA) showed reduced ability to inhibit the entry of HCoV-NL63, but lost ability to inhibit or enhance the entry of all other tested viruses (Zhao et al., 2014, 2018).

## Antiviral Activity of Human IFITM Variants

The antiviral activity of many naturally existing human IFITM variants has been tested in cell cultures. As shown in Table 1 and mentioned in previous sections, although SNP rs12252 is predicted to encode a splice variant specifying a N-terminally truncated isoform IFITM3 (Everitt et al., 2012), the putative Δ21-IFITM3 protein has not been detected in the tissues or cells of affected subjects (Wu et al., 2017; Makvandi-Nejad et al., 2018). However, subcellular localization and antiviral activity of genetically engineered Δ21-IFITM3 have been extensively investigated in cultured cells (John et al., 2013; Compton et al., 2016). Interestingly, IFITM2 was reported recently to have a similar N-terminally truncated isoform (Δ20-IFITM2) expressed in human innate immune cells and CD4^+^ T cells (Wu et al., 2017). Compared with the full-length wild-type IFITM2, Δ20-IFITM2 demonstrated an increased plasma membrane accumulation and enhanced antiviral activity against HIV-1, particularly, HIV-1 with CXCR4 tropism (Compton et al., 2016; Wu et al., 2017). In addition, expression and antiviral activity of some human nonsynonymous IFITM3 variants have also been investigated in cell cultures (John et al., 2013). While many of the SNPs results in undetectable levels of IFITM3 in the transfected cells, presumably due to the reduced stability of mutant proteins, and thus loss of antiviral activity against IAV, a few SNPs did not apparently alter the amount of IFITM proteins but impaired the antiviral activity against IAV. It will be interesting to test all the IFITM variants against a group of viruses and identify the SNPs that potentially impact the control and pathogenesis of specific viral infections in humans.

**Table 1:** Human IFITM variants and their biological functions.

Gene	Variant	Allele Frequency		Protein expression or steady	Subcellular distribution	Restriction of IAV	Restriction of HIV-1
IFITM3	rs34481144	All:	G:0.822; A:0.178	IFITM3 Expression is	Endosome and	Largely attenuated	Not available
		AFR:	G:0.957; A:0.043	downregulated	lysosome	Lower CD8^+^ T cells	
		AMR:	G:0.767; A:0.233				
		EAS:	G:0.994; A:0.006				
		EUR:	G:0.538; A:0.462				
		SAS:	G:0.793; A: 0.207				
IFITM3	Δ21 IFITM3/rs12252	All:	T: 0.764; C:0.236	Similar to wild type	Endolysosome and	Attenuated	Enhanced
		AFR:	T: 0.760; C:0.260		periphery of plasm		
		AMR:	T: 0.823; C:0.177		membrane		
		EAS:	T: 0.472; C:0.528				
		EUR:	T: 0.959; C:0.041				
		SAS:	T: 0.853; C:0.147				
IFITM3	H3Q/rs1136853	All:	C:0.961; A:0.039	Not available	Not available	Similar to wild type	Not available
		AFR:	C:0.948; A:0.052				
		AMR:	C:0.970; A:0.030				
		EAS:	C:1.000				
		EUR:	C:0.971; A:0.029				
		SAS:	C:0.920; A:0.080				
IFITM2	Δ20 IFITM2	Alternative transcription start site		Similar to wild type	Endolysosome and plasm membrane	Not available	Enhanced inhibition of X4 HIV-1

*AFR, African; AMR, Admixed American; EAS, East Asian; EUR, European; SAS, South Asian.*

## IFITM Variants and Diseases

### IFITM Variants in Human Populations

As a potent viral restriction factor, IFITMs play a pivotal role in limiting the infection by multiple viruses and any genetic variation affecting the IFITM expression or function might contribute to viral pathogenesis. Thus, natural variation in *IFITM* genes and its association with illness severity has been extensively investigated. Currently, a dozen of SNP in *IFITM3* have been reported, some of which may modulate IFITM expression, affect RNA splicing, or result in nonsynonymous or synonymous variants (Table 1). Several SNPs affecting function of IFITMs have been investigated for their association with morbidity, severity and prognosis of microorganism infection (Everitt et al., 2012; Shen et al., 2013; Zhang et al., 2013, 2015; Wang Z. et al., 2014; Allen et al., 2017).

The most studied SNP associated with severe outcomes of IAV infection is SNP rs12252, which is a nonsynonymous variation in the first exon of *IFITM3* (Everitt et al., 2012). The substitution of the major allele of T with alternative allele of C was predicted to alter IFITM3 mRNA splicing and generate a N-terminally truncated variant of IFITM3 with 21 amino acid residues deletion (Δ21-IFITM3) (Everitt et al., 2012). The SNP rs12252 has a higher prevalence in the population of East Asia than Europe (0.528 vs. 0.041) (Everitt et al., 2012). Moreover, the homozygous CC genotype and heterozygous TC genotype have significantly higher frequency in East Asia than that in Europe (0.300 vs. 0.000 and 0.456 vs. 0.082, respectively) (Everitt et al., 2012).

rs34481144 located at the *IFITM3* promoter is associated with severe influenza in three human cohorts (Allen et al., 2017). SNP rs34481144, wherein the majority G allele is replaced with a minor A allele, controls *IFITM3* promoter activity and determines the expression level. Compared to the G allele, A allele has activities of decreased IRF3 binding and increased CTCF binding, thus resulting in lower *IFITM3* expression. SNP rs34481144 has diverse allele and genotypic frequencies in different human populations (Table 1). The allele frequency of rs34481144-G is higher in the population of East Asia and Africa than that in Europe (0.957 and 0.994 vs. 0.538, respectively) (Allen et al., 2017). Also, the homozygous GG genotype has significantly higher frequency in East Asia than Europe (0.988 vs. 0.294) (Allen et al., 2017).

### IFITM3 Variants and Influenza A Virus Infection

Several groups previously reported that *IFITM3* SNP of rs12252 is associated with the susceptibility and severity of patients with seasonal influenza and pandemic H1N1 and H7N9 IAV infection (Everitt et al., 2012; Zhang et al., 2013; Wang Z. et al., 2014; Pan et al., 2017). As summarized in Table 2, Everitt et al. (2012) first discovered that a higher allelic frequency of rs12252-C exists in Caucasian hospitalized influenza patients than healthy control. Moreover, they observed a remarkably higher frequency of the homologous CC genotype in hospitalized influenza patients compared to the normal European population (5.7% vs. 0.3%) (Everitt et al., 2012). Importantly, another group reported that the minor C allele of rs12252 in the Caucasian population is much more prevalent in Han Chinese and Japanese and is associated with disease severity in patients with pH1N1/09 IAV infection (Zhang et al., 2013). In line with this observation, results obtained from study of H7N IAV infection revealed that H7N9 infected patients with rs12252-CC genotype exhibited accelerated disease progression and increased mortality rate than patients with the TC and TT genotype (Wang Z. et al., 2014). These studies collectively suggest that rs12252-C is a risk allele associated with severe IAV infection. However, several recent studies from European or Africa-American ethnic groups did not support the association of rs12252 with severe influenza infection (Mills et al., 2014; Lopez-Rodriguez et al., 2016; Randolph et al., 2017). The rare frequency of rs12252-C in European population may account for this discrepancy. Through meta-analysis of 11 studies, rs12252 T > C was associated with risk to severe influenza infection with odds ratio of 1.69 (95% CI 1.23, 2.33) in both European and East Asian populations, but for the mild infection, the results remained uncertain (Prabhu et al., 2018).

**Table 2:** Association of IFITM rs12252 alleles on viral pathogenesis in humans.

Study of rs12252		Allele Frequency		Genotype Frequency (%)			Allelic *P*-value (patients vs. Control.)
	Population	C	T	CC	TC	TT	
Influenza study by Everitt et al. (2012)	Welcome Trust Case Control Consortium 1 (WTCCC1, United Kingdom) (*N* = 2,938)	0.034	0.966	0.3	6.7	93.0	0.00006
	British hospitalized patients with seasonal influenza A or B virus or pandemic influenza A pH1N1/09 infection (N = 53)	0.094	0.906	5.7	7.5	86.8	
Influenza study by	Han Chinese 1000 Genomes (*N* = 197)	0.502	0.498	25.4	49.8	24.9	0.0001
Zhang et al. (2013)	All hospitalized pH1N1-infected patients (*N* = 83)	0.657	0.343	42.2	47.0	10.8	
	Mild Infection (*N* = 51)	0.559	0.441	25.5	60.8	13.7	0.0006
	Severe Infection (*N* = 32)	0.813	0.187	68.7	25	6.3	
Influenza study by	Chinese 1000 Genomes	0.445	0.555	26	37	37	<0.05
Wang Z. et al. (2014)	Hospitalized H7N9 influenza patients (*N* = 16)	0.59	0.41	37	44	19	
Influenza study by	GRACE healthy control (*N* = 2623)	0.035	0.965	0.15	7.7	92.1	0.753
Mills et al. (2014)	GAinS hospitalized patients with severe H1N1 infection (*N* = 34)	0.044	0.956	0	8.8	91.2	
Influenza study by	General Spanish population (*N* = 246)	0.0345	0.965	0.0	6.9	93.1	
Lopez-Rodriguez et al. (2016)	Patients with influenza virus infection (*N* = 118)	0.063	0.936	0.84	11.0	88.1	0.074
	Patients with mild influenza virus infection (*N* = 58)	0.069	0.931	1.7	10.3	88.0	0.11
	Hospitalized patients with primary viral pneumonia (*N* = 60)	0.058	0.942	0.0	11.7	88.3	0.171
Influenza study by	European 1000 Genomes (*N* = 503)	0.041	0.959	0	8.2	91.8	0.80
Randolph et al. (2017)	PICFLU-White non-Hispanic (*N* = 185)	0.038	0.962	1.1	5.4	93.5	
	African 1000 Genomes (*N* = 661)	0.26	0.74	7	38.1	54.9	0.97
	PICFLU-African American (*N* = 56)	0.258	0.742	7.1	37.5	55.4	
AIDS (Zhang et al., 2015)	Chinese MSM HIV-1-negative control (*N* = 196)	0.528	0.472	35.20	43.88	20.92	0.441
	Acute HIV infection with rapid progression in MSM cohort (*N* = 74)	0.608	0.392	29.73	62.16	8.11	
HFRS (Xu-Yang et al., 2016)	Han Chinese 1000 Genomes (*N* = 208)	0.522	0.478	26.92	50.48	22.60	0.0076
	Chinese patients with severe HFRS (*N* = 41)	0.683	0.317	43.90	48.78	7.32	

*P-value refers to the differences of allele frequencies between patients infected with different viruses and the control group (general population, healthy population or virus-negatives as used in the specific studies).*

Although the association of rs12252 with the susceptibility to influenza infection and disease severity was observed by many studies, the mechanism for this association is largely unknown. As mentioned above, although rs12252-C is speculated to encode a N-terminal truncated IFITM3 with attenuated antiviral activity to impede IAV entry, the truncated variant has not been detected so far (Everitt et al., 2012; Makvandi-Nejad et al., 2018). Moreover, the homozygous CC genotype was demonstrated to only express full-length IFITM3 at a similar level to TT genotype (Wu et al., 2017). Thus rs12252-C genetic variant does not appear to affect the biochemical nature and expression of IFITM3. Obviously, further investigation to determine whether rs12252-C influences IFITM3 gene splicing or protein levels in a cell type-specific manner and whether this SNP co-segregates with a different causative allele is warranted.

A recent study found that another *IFITM3* SNP rs34481144 has a strong association with disease severity in three influenza cohorts (Allen et al., 2017). rs34481144 is located in the promoter region of *IFITM3* (Figure 1). The substitution of the majority allele of G with minority allele of C reduces IFITM3 expression level and consequently results in the reduced number of antiviral CD8^+^ T cells in lung tissue upon IAV infection (Allen et al., 2017). In cohort FLU09, a cohort of naturally acquired influenza infection, a higher frequency of homozygosity of risk A allele was observed in patients with severe illness than the mild cases (33.3% vs. 1.3%) (Table 3; Allen et al., 2017). Also, an increased frequency of the A allele was found in patients who suffered with severe influenza in other two cohorts (Table 3; Allen et al., 2017). Thus, rs34481144-A is a risk allele associated with severe IAV infection and its association with other viral infection disease deserves to be investigated in future.

**Table 3:** Impacts of IFITM variant rs34481144 on viral pathogenesis in humans.

Study of rs34481144 with influenza (Allen et al., 2017)		Allele frequency		Genotype frequency (%)			Allelic *P-*value (severe vs. mild)
	Population	A	G	AA	AG	GG	
Cohort FLU09 (naturally acquired influenza infection) (*N* = 86)	Mild (*N* = 77)	0.169	0.831	1.3	31.2	67.5	0.037
	Severe (*N* = 9)	0.500	0.500	33.3	33.3	33.3	
Cohort of genentech challenge study (*N* = 42)	Mild (*N* = 23)	0.282	0.718	4.3	47.8	47.8	0.01
	Severe (*N* = 19)	0.553	0.447	21.1	68.4	10.5	
PICFlu cohort (pediatric influenza cohort of critically ill children) (*N* = 265)	Mild (*N* = 247)	0.333	0.667	14.1	38.3	47.6	0.047
	Severe (*N* = 18)	0.500	0.500	17.6	64.7	17.6	

*P-value refers to the differences of allele frequencies between mild influenza patients and severe influenza patients.*

### IFITM3 Variants and Other Viral Infections

In addition to influenza, SNP of rs12252 was also observed to have association with development of AIDS and Hantaan virus associated hemorrhagic fever with renal syndrome (HFRS) (Zhang et al., 2015; Xu-Yang et al., 2016). Zhang et al. (2015) reported that rs12252 is associated with rapid progression of AIDS, but not the susceptibility to HIV infection. Patients of AIDS rapid progressors had a higher frequency of rs12252 CC and CT genotype than non-progressors (Zhang et al., 2015). Compared with TT genotype, more patients with CC/CT genotypes were characterized with higher viremia level and more significantly reduced CD4 T^+^ cells (Zhang et al., 2015). In addition, the association of rs12252-C and homozygous CC genotype with disease severity in HFRS patients infected by Hantaan virus was reported recently (Xu-Yang et al., 2016). A higher allelic frequency of rs12252-C was observed in severe HFRS patients hospitalized than healthy Han Chinese controls (68.29% vs. 52.16%). Also, severe HFRS patients had a higher frequency of rs12252 CC than patients with mild HFRS and healthy Han Chinese. Along these lines, this group also reported that the viral titer detected in the plasma of HFRS patients with rs12252-CC genotype was more significantly alleviated than those with the TC and TT genotype (Xu-Yang et al., 2016). Thus, rs12252 C is a risk allele associated with severity of AIDS and HFRS, indicating that SNP rs12252 may have a profound impact on the pathogenesis of multiple viral diseases.

## Future Directions

IFITMs are a group of small fusogenic proteins that restrict the infection of broad spectrum of viruses by inhibiting the fusion of viral and cellular membranes. However, whether IFITMs inhibit hemifusion, the process whereby the outer, but not the inner, leaflet of the viral and cellular membranes merge, or the transition from hemifusion to pore formation remains to be rigorously determined. It is also important to note that IFITMs do not always inhibit virus entry, but may promote membrane fusion under selected conditions, such as in the case of HCoV-OC43 infection (Zhao et al., 2014). Moreover, our recent studies indicated that specific mutations can flip the biological activity of IFITMs, from inhibiting to promoting the infection of selected human coronaviruses (Zhao et al., 2018). Those later findings strongly suggest that the fusogenic activity of IFITMs might be bidirectional and could be regulated by viral and host factors. The elucidation of molecular mechanisms underlying IFITM-mediated innate immunity via modulation of viral entry into host cells as well as the negative imprinting of progeny virions (virions incorporating IFITMs display decreased infectivity) will open new avenues for future research. We have only just begun to appreciate the role of IFITM proteins in innate antiviral defenses. Genetic association studies of rare and common variants may explain population-specific and individual variance in susceptibility to common viral pathogens and identify specific IFITM – virus interactions. Expansion of genetic studies incorporating gene knock-down or knock-off screens, single cell transcriptomics, epigenetic modification, and targeted sequencing for different viral infections will provide needed insights into cellular mechanisms and pathways involved in IFITM-mediated host response to viral exposure and infection.

## Author Contributions

All the authors participated in the draft and revision of this review article.

## Conflict of Interest Statement

The authors declare that the research was conducted in the absence of any commercial or financial relationships that could be construed as a potential conflict of interest.
